# Beyond the self: The role of co‐regulation in medical students’ self‐regulated learning

**DOI:** 10.1111/medu.14018

**Published:** 2019-12-01

**Authors:** Derk Bransen, Marjan J. B. Govaerts, Dominique M. A. Sluijsmans, Erik W. Driessen

**Affiliations:** ^1^ School of Health Professions Education Maastricht University Maastricht the Netherlands; ^2^ Department of Educational Development and Research Faculty of Health, Medicine and Life Sciences Maastricht University Maastricht the Netherlands; ^3^ Research Group Professional Assessment Zuyd University of Applied Sciences Heerlen the Netherlands

**Keywords:** clinical education, development, professional, qualitative research methods

## Abstract

**Context:**

Medical students are expected to self‐regulate their learning within complex and unpredictable clinical learning environments. Research increasingly focuses on the effects of social interactions on the development of self‐regulation in workplace settings, a notion embodied within the concept of co‐regulated learning (CRL). Creating workplace learning environments that effectively foster lifelong self‐regulated learning (SRL) requires a deeper understanding of the relationship between CRL and SRL. The aim of this study was therefore to explore medical students’ perceptions of CRL in clinical clerkships and its perceived impact on the development of their SRL.

**Methods:**

We conducted semi‐structured interviews with 11 purposively sampled medical students enrolled in clinical clerkships at one undergraduate competency‐based medical school. Data collection and analysis were conducted iteratively, informed by principles of constructivist grounded theory. Data analysis followed stages of open, axial and selective coding, which enabled us to conceptualise how co‐regulation influences the development of students’ self‐regulation.

**Results:**

Data revealed three interrelated shifts in CRL and SRL as students progressed through clerkships. First, students’ CRL shifted from a focus on peers to co‐regulation with clinician role models. Second, self‐regulated behaviour shifted from being externally driven to being internally driven. Last, self‐regulation shifted from a task‐oriented approach towards a more comprehensive approach focusing on professional competence and identity formation. Students indicated that if they felt able to confidently and proactively self‐regulate their learning, the threshold for engaging others in meaningful CRL seemed to be lowered, enhancing further development of SRL skills.

**Conclusions:**

Findings from the current study emphasise the notion that SRL and its development are grounded in CRL in clinical settings. To optimally support the development of students’ SRL, we need to focus on facilitating and organising learners’ engagement in CRL from the start of the medical curriculum.

## Abstract 2

Is self‐regulated learning entirely self‐regulated? Bransen et al. share data demonstrating it to be embedded in co‐regulated learning, characterized shifts in regulatory focus, drivers for regulation, and co‐regulation partners.

## 1 INTRODUCTION

Self‐regulated learning (SRL) is considered a core competence of physicians and one that is essential to the safeguarding of patient care.^1^, ^2^ Many medical curricula therefore support medical students in developing SRL skills. Self‐regulated learning is generally described as a cyclical process, often triggered by the formulating of goals and the subsequent employment of strategies to achieve and monitor advancement towards those goals, followed by engagement in reflection and the formulation of new learning goals.^2^, ^3^ Research findings, however, indicate that students often struggle to regulate their learning in clinical learning environments as a result of the unpredictable, dynamic and messy nature of clinical workplace settings.^4^, ^5^

As early as 1989, the importance of environmental constraints and affordances to SRL was documented.^6^ Since then, the notion that medical students’ SRL is context‐dependent and socially derived has steadily gained momentum.^7^, ^8^ Learning climates of hospital departments, available learning opportunities and social interactions with others seem to influence students’ SRL,^9^ and medical trainees variably use SRL strategies, depending on social and contextual factors.^10^ A study by Berkhout and colleagues, for example, showed that novice students interact with peers and residents to help them formulate learning goals.^11^ Elsewhere, paediatric residents have been found to benefit from interacting with supervisors when pursuing goals.^12^ Similarly, surgical residents actively employ different strategies to engage their supervisors in the monitoring of performance during surgery.^13^

The potential impact of social interactions on medical students’ SRL is embodied in the concept of co‐regulated learning (CRL). In CRL, learners regulate their cognitions, motivation and behaviour together with other individuals in the environment.^14^ Essential to CRL are social interactions between students and others in their networks, through which learning processes, such as SRL development, are mediated or distributed.^15^ The concept of CRL reflects sociocultural learning theories that emphasise that learners are continuously influenced by their environment when simultaneously co‐producing and co‐creating the contextual surroundings in which they learn and work.^16^ By jointly engaging in learning activities, students and co‐workers in health care settings mediate one another's metacognitive and cognitive activities, facilitating or constraining individuals’ development and display of independent SRL behaviours.^17^ Findings from a recent study show that medical students’ engagement in SRL changes over time and is influenced by their perceptions of the roles of, and interactions with, others in the workplace setting.^11^ This illustrates that processes of CRL may influence the development of students’ ability to self‐regulate.

Examining how CRL, with social interactions as its main element, influences SRL development in workplace‐based learning environments will contribute to a deeper understanding of how students actually develop SRL in clinical settings. This may help to create clinical learning environments that effectively foster SRL development, adequately equipping future physicians for lifelong learning. However, research focusing on the developmental aspect of SRL and how social interactions influence medical students’ SRL development is still scarce. The present study aims to address this gap. Specifically, we aim to explore undergraduate medical students’ perceptions of CRL in clinical clerkships and its perceived impact on the development of their SRL.

## 2 METHODS

### 2.1 Methodology

We used a qualitative approach to explore medical students’ perceptions of relationships between CRL and SRL in clinical clerkships. Data collection and data analysis were conducted iteratively, informed by principles of constructivist grounded theory.^18^, ^19^ Using constructivist grounded theory, we acknowledged the research team's theoretical (DB, EWD, DMAS, MJBG) and practical (DB, EWD, DMAS, MJBG) backgrounds in SRL and CRL, and data collection and data analysis were informed by key concepts in SRL and CRL.

### 2.2 Setting

We conducted this study in the undergraduate programme (Masters degree) in medicine at Maastricht University, the Netherlands. The Masters programme comprises 3 years of clinical clerkships lasting between 12 and 22 weeks, in various hospital departments in an academic hospital and affiliated teaching hospitals. The Masters programme is designed according to the principles of competency‐based medical education, using the roles of the Canadian Medical Education Directives for Specialists (CanMEDS) as its overarching framework.^20^ Students’ SRL is supported throughout the programme by a portfolio and a mentor. Daily workplace supervisors are assigned to support and guide student learning in various workplace settings. Before the start of each clerkship, students formulate a learning plan that describes their personal learning goals and discuss this with their mentor and the supervisors assigned for that clerkship. At the end of each clerkship, students collaborate with their mentors to reflect on their experiences and the feedback uploaded to the portfolio and formulate new learning goals for the next phase of training. Learning plans are included in students’ portfolios, as are assessment data (eg feedback forms and test results), reflections on experiences, and any information students deem valuable for monitoring their personal and professional development.^21^

### 2.3 Participants and sampling

Overall, 79% of students in the Masters programme are female. We purposively sampled medical students enrolled in the Masters programme to ensure variety in year of clinical training and gender (Table 1). Students were invited to participate via e‐mail. Reminders were sent 2 weeks after the initial invitation. After 10 interviews, we considered that we had achieved data saturation and had gained an adequate understanding of the constructed themes and their relationships. Participants received a gift voucher for €10.

**Table 1:** Participant characteristics (*n* = 11)

	Students, *n* (%)
Gender	
Male	2 (18%)
Female	9 (82%)
Year of clinical training	
First year	2 (18%)
Second year	5 (46%)
Third year	4 (36%)
Age, (year), mean (range)	23 (21‐24)

### 2.4 Data collection

Between February and June 2018, the first author (DB) conducted semi‐structured interviews that lasted between 50 and 75 minutes. The research team developed an interview guide informed by key concepts of SRL, such as goal formulation and strategy design principles, monitoring and self‐reflection processes, as well as CRL (relevant others with whom students engaged, as well as the nature of their interactions). After obtaining informed consent, first author (DB) provided students with a description of SRL. The first part of the interview then aimed to obtain the student's perspectives on how he or she engaged in SRL in the clinical setting; the second part focused on the student's perceptions of the impact of social interactions on the development of SRL skills. The final part of the interview focused on developmental aspects of SRL and CRL (Appendix S1). During interviews, the interviewer prompted students to provide detailed examples and explanations of behaviours they described. The interview guide was updated iteratively between interviews, informed by concurrent data analysis. All interviews were tape‐recorded and transcribed verbatim.

### 2.5 Data analysis

Data collection and analysis followed an iterative process, allowing data collection and data analysis to inform each other. The first author (DB) and a research assistant (CN) independently read and coded transcripts 1‐3 line by line to capture concepts related to CRL and students’ development of SRL.

In the initial coding scheme, codes described various SRL and CRL behaviours such as the formulation of learning goals, asking for feedback and feedback reception, and details of the significant others with whom students engaged in CRL processes. Also included were factors related to and influencing the development of SRL such as the phase of clinical training. Coding frameworks were compared and discussed until consensus was reached. DB used the coding scheme to code transcripts 4‐11, continuously refining the coding framework through constant comparison and discussion of findings with the research team. The research team subsequently categorised codes and identified relationships between themes through axial coding. During the final stage of analysis, the research team used selective coding to develop theory about the influence of CRL on students’ SRL development. To facilitate this process, DB constructed concept maps, presenting themes, sub‐themes and their relationships, which were used during research team meetings to guide theory development. DB furthermore captured reflections, ideas and interpretations in memos throughout the data analysis. Data analysis was supported by the qualitative research software atlas.ti (ATLAS.ti Scientific Software Development GmbH, Berlin, Germany).

### 2.6 Reflexivity

Within our methodological approach, meaning is constructed through interactions with research participants, the data generated, and their interpretation by the research team. DB is a PhD candidate with an educational background in work and organisational psychology. MJBG and EWD are medical educators and researchers involved in the development of the assessment aspect of the Masters programme at Maastricht University. DMAS is an education researcher whose main areas of expertise are professional assessment, curriculum design and student involvement in assessment practices. Maastricht University is characterised by its problem‐based learning (PBL) philosophy, in which collaborative and self‐directed learning play pivotal roles. EWD and MJBG have worked within this setting for substantial lengths of time and DB has had formal education according to PBL principles; these backgrounds may have influenced their views on CRL and SRL and how these may be related. DB used memos and field notes to capture views and interpretations throughout data collection and analysis.

## 3 RESULTS

From the data, we were able to construct three major shifts in students’ SRL, which were simultaneously influenced by and exerted influence on students’ engagement in CRL as they progressed through clerkships. Firstly, students expressed a shift with regard to whom they involved in CRL, bestowing an important role on peers at the start of clerkships and increasing their preferences for clinician role models towards the end of the programme. Secondly, novice students’ SRL was mainly externally driven by cues in the environment, whereas experienced students’ SRL was increasingly driven by intrinsic cues and self‐motivation. Finally, regulatory behaviours shifted from a task‐oriented approach towards a more comprehensive approach that focuses on professional competence and identity formation. We will detail these shifts and the influences of social interactions between students and relevant others that contribute to the shifts.

### 3.1 From interacting with peers to interacting with clinician role models

Students described a change with regard to whom they engaged with in CRL over the course of clerkships. At the start of the programme, interactions with peers played an important role, serving several goals. Novice students indicated being unsure of clerkship expectations and opportunities and thus relying on one another to develop an understanding of the new learning environment:

What I also really like when you're new to a place is that other interns [fellow students] always take you along, show you the way, and also point out how you can best tackle this here. (Student 2, Year 2)

Additionally, novice students tended to rely on one another to create a frame of reference in order to formulate learning goals, and to think about learning strategies and selection of learning opportunities:

You have a frame of reference, more or less. Like, you [fellow students] do it like this or think that is important or you already know it. Well, maybe I should know it too, so how do you do it? (Student 1, Year 2)

Interactions with peers thus exposed gaps in students’ knowledge or skill proficiency, helping them to formulate goals and strategies to address these gaps.

Although peer interactions helped novice students to navigate and feel more comfortable in the new learning environment, students gained experience in adapting to new environments with each new clerkship. Over time, they participated increasingly in health care tasks, which helped them to develop a firmer grasp of what they wanted or needed to learn. Experienced students reported that they then relied on other sources to help regulate their learning, referring to clinician role models such as attending physicians and residents. These role models influenced students’ SRL behaviour through the provision of feedback, but especially through the stimulation of self‐reflection based on interactions with and observations of these physicians:

In principle, the physician is your role model for 3 years, so you observe him and think, how would I do that and do I want to be this kind of doctor or do I want to be another kind? And through meeting all these different specialists, you create your own, let's say, [idea of the] physician you want to become. (Student 9, Year 2)

### 3.2 From external regulatory drivers to internal regulatory drivers

At the start of the programme, students’ SRL seemed to be largely externally driven. Novice students described complying with portfolio instructions and relying on input from others when formulating learning goals prior to a clerkship. Quite often, however, they also found that these learning goals were not well aligned with learning affordances in a specific setting, which led to feelings of detachment from their learning plans. During the first clerkships of the programme, students then depended on others in the actual workplace setting to help them reactively adjust learning goals and strategies. Residents and physicians, for example, helped students regulate their learning by providing clear and explicit directions on what they thought were important learning goals:

Yes, of course if a doctor says, ‘Hey, this is fun to do’ or ‘You should be able to do this by the end of the clerkship,’ then of course, you go along with it. (Student 11, Year 1)

Workplace supervisors in particular seemed to play important roles in this respect:

You always chat with your workplace supervisor, and they say things like: ‘We expect you to learn this and that, and get this and that out of your clerkship.’ If you haven't already factored these [expectations] into your goals beforehand, then you think, okay, I'll take them into account [now]. (Student 10, Year 1)

Self‐regulated learning was also often grounded in situations in which a student and a physician were cooperating in a joint activity. Students reported that some physicians consistently posed questions about the activity, which uncovered gaps in knowledge or skill proficiency. This helped students to direct their learning activities and processes and bridge the gaps:

When you're in the operating room, and the surgeon asks six questions on anatomy and you can only answer one, then you sure know you have something to do at home. (Student 9, Year 2)

Novice students’ SRL thus seemed to be predominantly initiated by others in the learning environment.

Over time, as students gained a better understanding of what was expected of them, they increasingly began to think about the type of physician they wanted to become and developed social sensors that helped them look for and select suitable others to provide input for their learning. Having experienced a variety of health care settings, and as they became increasingly aware of their developing professional identity, experienced students seemed better able to take the initiative in engaging others in CRL. They seemed increasingly willing and able to ‘take charge’ of regulatory activities. Appreciating their personal learning needs, they seemed more confident in actively asking relevant others to help them regulate their learning:

Then once I really did ask, on [the clerkship in] internal medicine, if there is a difficult conversation, or there's bad news to deliver, please let me do it because it's my learning goal. (Student 6, Year 3)

Students reported that the extent to which they were able to engage in CRL to support their learning depended on whether they were willing and sufficiently confident to use SRL skills such as goal setting and the devising of strategies to achieve these goals. That is, students explained that when they took control of their learning based on a firm grasp of what they wanted to learn, they more readily found the courage to engage and mobilise others in the environment to select or create learning opportunities:

I do feel that I can control it [my own learning] a lot. By taking the initiative and showing that you want to learn. And also having the courage … to ask questions and to ask for things to do, or to come up with suggestions. (Student 1, Year 2)

### 3.3 From specific tasks to professional competence and professional development

Students indicated that the focus of the regulation of learning shifted over the course of clerkships. Novices described being task‐oriented. That is, goal setting was characterised by high levels of granularity; novices focused strongly on attaining clearly demarcated learning goals (and on *formulating* learning goals):

I look at the framework plan for physicians [CanMEDS framework]. It's subdivided into competencies and those sub‐competencies are divided into specific components. (Student 10, Year 1)

For novices, CRL thus mainly entailed engaging others (peers and mentors) in formulating and reflecting on narrowly defined, task‐specific learning goals. Consequently, self‐reflection most often consisted of an evaluation of whether or not formulated learning goals were attained. Experienced students reported that, as they gained experience by progressing through clerkships, they not only became more capable of formulating realistic and feasible learning goals, but they also shifted the focus of their learning goals on to more comprehensive aspects of medical expertise. That is, experienced students formulated learning goals that transcended the high level of granularity applied by their novice counterparts, and adopted a broader approach to goal setting and self‐reflection. Thus, the learning goals of experienced students rose to the level of professional development and identity formation:

In your first clerkship, you are more concerned with specific learning goals, to improve things, and in your last clerkship you are more concerned with how do I become a good doctor in general. Then I'm no longer bothered by the specifics […] I'm more concerned with the kind of doctor I want to be, what specialism I want to work in, and how I can direct my choices to achieving that. (Student 6, Year 3)

## 4 DISCUSSION

This study aimed to explore medical students’ perceptions of CRL in clinical workplace settings and to investigate how interactions with others in the learning environment affect the development of students’ SRL. Our findings show that students’ engagement in CRL and SRL changes over time, and is influenced by changing perceptions and experiences of the learning environment and the development of a professional identity. Our findings suggest that three major shifts are key to students’ development over time: (a) a shift in the selection of CRL partners, which moves from peers to clinician role models; (b) a shift in SRL behaviours, which tend to be externally driven in novice students and become internally driven and involving of the deliberate, proactive engagement of others in more experienced students; and (c) a shift in regulatory focus as learners move from well‐defined tasks and task requirements to a broader view of professional competence in relation to the developing of a professional identity.

The findings of our study corroborate those of previous research and stress the importance of social interactions in clinical settings for the development of medical students’ SRL. Aligned with findings from the study by Berkhout and colleagues,^11^ the current findings suggest that the influence of others on students’ SRL differs between novice and experienced students. Whereas novice students mainly rely on peers to co‐create a frame of reference to help them navigate the learning environment, experienced students learn to take control, to interact with clinician role models as more experienced others and to increasingly engage these in co‐regulatory activities to support their own learning. Participants furthermore reported that they could more readily engage others in their learning if they had developed a clear view about what they wanted to learn and how the achievement of these goals would contribute to the development of professional competence. These findings suggest relationships between SRL and CRL are reciprocal: SRL skills develop through interactions with others, whereas the mastery of SRL skills increasingly enables students to proactively engage in meaningful CRL to enhance their learning and competence development. Active participation in health care tasks, as well as reflection on the type of physician the individual wants to become, seem to be important facilitating factors, influencing students’ development into learners who are increasingly able and willing to regulate their learning through interactions with others in the work and learning environments.

Participants in our study clearly indicated that the ability to regulate their own learning depended on their having a deep understanding of the clinical setting as a learning environment in which learning and work are intertwined. Students repeatedly reported being challenged as well as frustrated when tasked with setting learning goals if their understanding of the learning opportunities within clerkships was insufficient. In consequence, students felt they were then forced to continuously adjust and refine their goals based on observation of and negotiation with residents, physicians and peers, as well as on actual learning and work experiences. These findings are in line with those of previous studies which showed that goal setting in clinical contexts requires negotiation between students and supervisors and engagement from supervisors if goals are to be realistic and attainable.^22^, ^23^

Our findings also showed that the extent to which and the pace at which students are embedded in teams in clerkship environments influenced their ability to proactively engage others in the regulation of their learning through the solicitation of feedback or by negotiating their own active participation in health care tasks. This may suggest that we need to facilitate the prolonged inclusion of students into communities of practice (CoPs) to capitalise on the opportunities for CRL in specific work settings.^24^ In CoPs, individuals typically engage in learning from and with each other by sharing goals and having conversations about how best to achieve these goals, and thereby continuously deepening one another's knowledge and skills.^25^ Not only do CoPs create opportunities to engage peers and more experienced others in the regulation of learning, but they also offer opportunities to engage in discussions about the quality of work and health care, stimulating students’ reflections on what it takes to be and become a medical doctor and thereby supporting their professional identity formation and subsequent development of SRL. Based on the findings of our study, as well as findings from previous research,^11^ peers seem to fulfil important roles in novice students’ development of SRL because they can support students in goal setting and familiarise newcomers to opportunities within and expectations of the learning environment. Peer‐assisted learning (PAL) has furthermore been shown to contribute to students’ professional identity formation.^26^, ^27^ We therefore may need to pay more attention to stimulating PAL within CoPs, by, for example, forming learning and working partnerships between novice and more experienced students. As PAL entails bidirectional support,^28^ stimulating PAL within CoPs will deliver the added benefit of developing learners’ coaching and teaching skills.^29^

Overall, our findings support the notion of SRL as being embedded in CRL, an idea gaining momentum.^30^ The current study highlights the need to look ‘beyond the self’ when examining and facilitating students’ SRL development. Our findings reflect essential notions within sociocultural learning theories, indicating that workplace learning, including the development of SRL, always occurs in interactions with others, and that students’ SRL always involves engagement in CRL. Rather than developing into students who learn ‘autonomously’ and ‘independently’ from others, students’ transformation into self‐regulated learners is reflected through significant changes in the way that they engage in CRL and their increasing ability to purposively and meaningfully engage others in their learning to support their development of competence. Although it is increasingly acknowledged that SRL is context‐dependent, research often still emphasises and underscores learner independency and autonomy.^31^, ^32^ The present study challenges this idea and instead advocates for considering SRL, as well as SRL development, as being embedded in social interactions in clinical workplace settings. Conceptualising the development of students’ SRL as the development of students’ ability to engage in CRL in CoPs has important implications for how we organise clinical clerkships and how we educate medical students to regulate their learning. For instance, we may need to reconsider current models of medical training in which students rotate through multiple short‐block rotations. Rather, we should facilitate the development of safe and trusting longitudinal relationships within health care teams in longitudinal integrated clerkships,^33^ for example^33^ to allow students to move towards the centre of the CoP and to fully capitalise on opportunities for CRL. Furthermore, in order to help students develop into learners who are able to regulate their learning in workplace settings, we may not only need to offer training and coaching in skills such as goal setting, self‐assessment and reflection, but first and foremost to pay attention to the skills that enable students to engage in CRL, such as feedback seeking and engaging others in learning conversations. Acknowledging from the outset of medical training that SRL is embedded in CRL may assist us in better preparing students to optimise their learning in workplace settings, and may help medical education and research to truly move beyond the self.

### 4.1 Limitations

This study was conducted with a limited sample of 11 participants. Most (nine students) were enrolled in the second or final years of clinical training. This means that these students were able to describe their behaviour and thoughts at the start of clinical training only retrospectively, which may have made them susceptible to memory bias. Co‐regulated learning and SRL are abstract theoretical constructs, which students may have found difficult to talk about in practical terms. In constructing the interview guide, the research team attempted to formulate the questions as concretely as possible. It may be that CRL is more prevalent than students realise, and much of CRL may be hidden or implicit. For example, some students indicated after the interview had finished that they did not usually give very much thought to the concepts discussed in the interview. In a similar vein, participants were self‐selected volunteers, which may imply that only students who actively thought about their learning agreed to participate.

## 5 CONCLUSIONS

Our study emphasises the idea that SRL and SRL development are embedded in social interactions and CRL in clinical workplace settings. We can support medical undergraduates’ development of SRL when CRL is structurally embedded in clerkships, acknowledging that the ability to self‐regulate implies the ability to engage in meaningful interactions with others to support learning processes. In order to facilitate medical students’ transformation into lifelong learners, we therefore need to support and organise learners’ engagement in CRL. For competency‐based medical education, this means that we should reconsider the ways by which we include medical students in CoPs in order to encourage workplace partnerships between novice students and more experienced students and health care professionals. By capitalising on the opportunities for CRL in clinical settings, we may be able to better equip students with the learning skills they need to develop into health care professionals who are able to optimise their learning and provide high‐quality care in rapidly changing health care systems throughout their professional careers.

## CONFLICTS OF INTEREST

None.

## AUTHOR CONTRIBUTIONS

DB is the principle author of the work. All authors contributed to the conception and design of the work. DB collected the data and drafted the initial manuscript. All authors were involved in data analysis and interpretation, and contributed to revisions of the paper. All authors approved the final manuscript for publication.

## ETHICAL APPROVAL

This study was approved by the Dutch Society for Medical Education (Nederlandse Vereniging voor Medische Onderwijs [NVMO]) Ethical Review Board (NVMO ERB ref. 970).
