# Supplementary material for: Beyond the self: The role of co‐regulation in medical students’ self‐regulated learning
Source: Med Educ. 2019 Dec 1;54(3):234–41. doi: 10.1111/medu.14018 (PMC7065189; doi:10.1111/medu.14018)
Supplement: Supplementary file 1 [file MEDU-54-234-s001.docx]

**Appendix A Interview Guide**

**Demographics**

- Name;
- Age;
- Which clerkship are you currently in?
- Which educational year are you currently in?

**Learning**

- Can you give a description of the way you learn in the workplace during clerkships?
- Which factors help and hinder your learning during clerkships?
  - How do these factors help and hinder?
- What role do others play in your learning during clerkships?
  - Who plays a role?
  - How do they play their role(s)?

**Self-regulated learning**

- To what extent do you have the idea that you are able to give direction to what you learn in the clerkships and how you learn that?
- How do you give direction to your learning (activities) in clerkships?
- What is the value to you of being able to give direction to and maintain control over what you want to learn and how you learn that?

**Co-regulated learning**

- How do others in clerkship environments help you in how you give direction to what you want to learn and how you learn that?
  - Who help you?
  - How do they help you?
  - With what do they help you?
- How do others in clerkship environments hinder you in how you give direction to what you want to learn and how you learn that?
  - Who hinder you?
  - How do they hinder you?
  - In what are you hindered by them?
- How do you seek help from others in clerkship environments with regard to giving direction to what you want to learn and how you learn that?
  - Who do you seek help from?
  - How do you look for help?
  - What are you looking for help for?

**Development**

- Has the way in which you give direction to your learning changed during your clerkships?
  - How?
  - What do you think that caused this change?
- Has the way in which you seek help from others with regard to giving direction to your learning changed during your clerkships?
  - Did you seek help from other people at the start of your clerkships than you do now?
  - What do you think that caused this change?
- Has the way in which others help you in giving direction to your learning changed during clerkships?
  - Do you receive help from other people now than you did at the start of your clerkships?
  - How does the help you receive from others differ?
  - What do you think that caused this change?
